# Tri-State Medical Society of Alabama, Georgia and Tennessee — Seventh Annual Meeting

**Published:** 1895-12

**Authors:** 


### Society Notes.

### Tri-State Medical Society of Alabama, Georgia and Tennessee—Seventh Annual Meeting.

FIRST DAY.

President R. M. Cunningham, presiding.

Session opened with prayer by J. W. Bachman.

After the transaction of miscellaneous business the president delivered his address, entitled

TUBERCULOSIS, based on a large experience in the penitentiary of Alabama. The paper showed a wonderful predisposition of the negro race to tuberculosis, compared to the whites, which he explains as follows:

1. The negro has acquired, since his emancipation, a predisposition to tuberculosis, which is hereditary.

2. A greater liability to diseases which produce a local predisposition to tuberculosis, especially bronchial and intestinal catarrh and pleurisy.

3. He is physically and mentally and morally inferior to the white man; therefore,

4. He is more liable to contract disease, particularly tuberculosis and thoracic diseases (local and from infection) generally.

5. His changed social, religious, political and industrial relations, involving, as a rule, a change from the segregate to the aggregate, from country to town and from farm to public works.

6. His disregard for all rules of sanitation.

The greatest mortality was between twenty and thirty years.

In two prisons at Pratt City, a mile apart and under the same management and hygienic conditions, there were at No. 1 forty-five deaths from tuberculosis, at No. 2 twenty-six deaths. Explained by the fact that there was an epidemic of diarrhœa at No. 1, proving that intestinal catarrh predisposes to tuberculosis of the peritonial form. The excess of mortality at No. 1 over No. 2 being nineteen, exactly the number which died from tubercular peritonitis. He always found an epidemic of diarrhœa followed by a large number of cases of tubercular peritonitis.

The causes of tuberculosis he defined to be, 1st, Essential, tubercle bacillus; 2d, The predisposing, (a) an inherited constitutional predisposition, (b) an acquired constitutional or local predisposition. The predisposing *never alone* produces the disease, the essential rarely. Primarily, it is the result of hetero-inoculation, and, as a rule, is a local infection, producing chronic local changes the general and acute forms being due to auto-infection.

In general acute miliary tuberculosis the lungs, peritoneum, spleen, sometimes the liver, rarely the kidneys, were involved, the patient dying before the caseous stage of the disease set in. These cases follow pleurisy frequently, notably after a large amount of fluid was withdrawn and from tubercular glands. In the chronic general form the process involves the same organs, but the infection is not so general and the caseous stage is often reached and local inflammatory lesions generally found. In both there may be an active tubercular inflammation developed in some organs rapidly terminating the disease. The bacilli were distributed by the circulation.

The acute forms are generally the result of auto-infection from a chronic tuberculosis which may not have been suspected. The acute forms very rare. The bone rarely affected. The one most frequently involved being the sternum.

H. Berlin thought that the reason the disease prevailed so largely in prisons, was 1st, because the convicts were generally in a weakened condition; 2d, while the buildings were apparently clean, still they contained the germs, but the most important was the fact that the dust inhaled, which shows sharp corners under the microscope, cut the delicate tissue and admitted the bacilli.

C. Holtzclaw had noticed the frequency of tuberculosis in the county institutions. While the left lung was first affected in the white, it was the right in the negro. The greater liability of the negro to the disease was due to the fact that the white race had acquired an immunity from long contact.

J. B. Murfree could not endorse that diarrhœa had anything to do with inducing the disease, except as it weakened the patient. While the bacillus was the cause, an equally important factor was the predisposition.

J. B. Cowan thought we could lift up the vital energy and put the functional activity in such a condition as to resist the disease.

G. A. Baxter differed from Dr. Murfree in regard to diarrhœa as a factor in the production of the disease. It acts by allowing an entrance for the bacilli. One agent the writer did not mention was the food, and especially milk. In China, where the disease is unknown, it is attributed to the non-use of milk. He had frequently seen tuberculosis in bones and in glands of the neck. He could see no objection to the removal of the latter. An imperfect removal might result in general infection, but where there are two or three, they could be thoroughly removed.

P. L. Brouillette regretted that the microscope had not been used by the writer, as it was sometimes impossible to diagnose tuberculosis.

Dr. Cunningham, in closing the discussion said that dust produced a fibroid phthisis, but did not produce tuberculosis, but might predispose to the disease. He did not find typical tuberculosis in the negro. Believes, he is acquiring a predisposition to tuberculosis. Both lungs were affected as a rule. The diarrhœa predisposed to the disease by abrading the membrane and giving entrance to the baccilli. The weakness of the paper was that the microscope was not used, but the clinical history and the gross lesions were enough to complete the diagnosis.

G. Manning Ellis, of Chattanooga, read a paper,*

PSEUDO-HYPERTROPHIC MUSCULAR PARALYSIS, and presented a patient giving the characteristic history. The boy was late in attempting to walk. The muscles were large, and seemed well developed. The gait was oscillating, the child,

---

*Published herewith.—Ed.

while seeming healthy and well developed, showed an increase in the difficulty of locomotion. The characteristic symptoms were shown. The peculiar gait, the lordosis, the manner of rising by placing the hands on the knees and then on the thighs, climbing up as it were with his hands, which is almost pathognomonic, the absence of the tendon reflex, and the diminution of the muscles of the leg which comes after the enlargement. The upper extremities showed the enlarged infraspinatus and the decrease in size of the latissimus dorsi, producing an absence of the axillary fold.

Willis F. Westmoreland asked if there was any adherent prepuce. The reply was that there was not. He reported two cases, in which there was adherent prepuce in one; there was an operation in the late stage, but with no improvement. He thought that if there were irritation from this, that an operation before the disease began, might be of benefit.

NIGHT SESSION.

A reception was tendered the Society at the Southern Sanitorium by Dr. and Mrs. R. P. Johnson.

---

SECOND DAY.

J. B. Murfree, of Murfreesboro, Tenn., read a paper on

THE PLACENTA: HOW AND WHEN DELIVERED.

He thought as soon as the child was born, Crede's method should be employed. If the placenta does not come away in twenty minutes, gentle traction should be made on the cord. Undue force should not be used. If this does not succeed, and especially if the placenta presents centrally, the hand should be introduced and the edge freed from its attachments. If the placenta was delivered as soon as the child was born, it would leave the mouths of the vessels open. Exactly when to remove it, can not be definitely stated in every case. Most practitioners wait too long.

R. E. Kime took issue with the author in regard to introducing the hand into the uterus, as that would add greatly to the danger of infection, and was rarely necessary. By wrapping the cord around the fingers of the right hand and pressing back the posterior of the cervix the placenta will slip out. He protested against the use of ergot, which contracted the os and prevented the delivery of the placenta.

W. G. Bogart delivers the placenta as soon as he ties the cord, gives the child to the nurse and prepares his hands, which takes about twenty minutes. He grasps the fundus and squeezes the placenta out, if possible; if not successful he introduces two fingers into the uterus and makes a rotary motion, with the other hand still on the fundus. Never makes traction on the cord, which he deems dangerous. Ergot should not be given to expel the placenta. The important point is to get all the placenta away. He has no fear of putting the hand into the uterine cavity if it was thoroughly cleansed, nor does he deem it necessary for this reason to use an intra-uterine douche.

Preston Scott thought that we were all too hasty to get rid of the placenta. Time should be given for tonic contraction. Gentle traction should be made on the twisted body of the placenta. Had excluded ergot from his practice entirely. He used the hot water douche to promote contraction.

J. P. Stewart waits longer than twenty minutes; an hour, if there was no hemorrhage. If the placenta was in the vagina, delivers at once, as it acts as a foreign body, causing contraction and tearing the membranes.

J. B. Cowan thought there was a happy medium between twenty minutes and an hour. He assists nature during the pains. If necessary puts his hand into the uterus. Post partem hemorrhage can be prevented by steady pressure on the fundus, kept up an hour, if necessary. Never pulls the cord.

Dr. Murfree said that ergot was liable to contract; hourglass contraction. There can be no harm in introducing the hand into the uterus.

E. H. Sholl, of Birmingham, read a paper, entitled

REFLECT.

Reporting cases which were not relieved because not properly investigated. A case treated for liver disease was relieved by treatment indicated by an examination of the urine, one diagnosed as consumption, relieved by a similar course, one of headache was found due to diabetes.

In these cases no examination of the urine had been made. Doctors as a rule should study their cases more.

J. B. Cowan said that the lesson of the paper was that all should learn to think. In this respect he found fault with modern education which was too much a process of cramming.

Willis F. Westmoreland thought that the modern medical college taught that every patient should be examined as well as if for life insurance. The day when albumen could be seen by looking at the bottle was passed. No college of any pretensions did not teach examination of urine and the use of the microscope. The modern student was better qualified than were those of the past.

J. B. Murfree thought the point of the paper was that we did not take time to think about our cases, not that we did not know how to make these examinations. The facts were not properly put together.

Preston Scott was surprised that in many cases seen in consultation, no examination of the urine had been made and the cases not properly studied.

W. C. Townes asked the writer what he meant by a rigid diet.

Dr. Sholl said that he meant the exclusion of all starchy foods, the use of meat, milk, eggs, oysters, cheese, turnips greens and such articles.

Willis F. Westmoreland presented:

### REPORT OF CASES: (a) TRACHEOTOMY FOR FOREIGN BODIES; (b) CYSTOTOMY FOR STONE.

He reported twenty-seven successful cases of tracheotomy for foreign bodies. In all the first ring was cut and the opening enlarged downward as necessary. Operated as early as possible. The external incision was made large. Muscles were separated, the fascia divided, vessels were ligated so that no forceps were in the road. If necessary, the isthmus of the thyroid was divided and ligated. No tenaculum was used in the trachea. Instead of trachea forceps the cut was held open with the silk thread introduced with a needle having no cutting edge. The foreign body was not probed for but the wound left open if necessary, in one case (cockle burr) for three days. The mucus will sometimes prevent the closure of the wound.

In closing the latter, the tissue under the mucus membrane is brought together with silk, using a needle having no cutting edge and the Halsted or mattress suture. Thus the layers are all brought together.

In cystotomy for stone he avoids rectal dilatation. To distend the bladder he uses hydrostatic pressure raising the water two feet. This causes the bladder to bulge through the opening. No tenaculum is used to steady the bladder but it is held with two artery forceps between which the opening is made as high as possible. After removing the stone and flushing the bladder the opening is closed with the Halsted suture. The closure is tested by raising the water for an instant three feet.

G. A. Baxter endorsed the position of the author. He was attached to the medio-lateral operation. Stone is rare in this section, there not having been a dozen operations in fifteen years. The disease is more frequent in Middle Tennessee. He advocated drainage in the supra-pubic operation.

J. A. Goggans related a case in which the larynx at the site of the thyroid cartilage was almost occluded by a new growth so that he had to operate rapidly to save the patient. Throwing the head back increased the difficulty of breathing, and the head had to be held forward and the larynx steadied with a tenaculum. He said that the supra-pubic was the operation par excellence for stone. He was prejudiced against the low operations as he was once called on to sew up a large fistulous opening between the bladder and the rectum in a case sent him. He had attempted to repair this opening by silk worm gut operating through the rectum. At the third operation he had divided the sphincter ani muscle and almost closed the fistula.

W. E. B. Davis said that he was glad that both operations were fairly considered. The results of the older surgeons who performed the low operation were good. He found that statistics of the supra-pubic operation were bad because bad cases were selected for that operation. A distinguished surgeon had so operated to save his statistics for the low operation. This shows how statistics can be doctored. He thought it well to leave the wound open for drainage, for there is always disease of the bladder and we may have a new stone in a few weeks.

R. J. Trippe said that his experience was limited to a few cases operated on for disease and advocated leaving the wound open for drainage. As a guide he used a sound and did not fill the bladder but always washed it out before the operation. In tracheotomy he used long scissors forceps and did not stop to ligate the vessels. These held the wound open and saved one assistant.

Dr. Westmoreland in closing the discussion said that he used no instrument to hold the trachea open as that took some room while the silk did not. Time was an important item, but we always have two minutes after the child quits breathing, as he can be resuscitated after that. He advocated cutting high in the subra-pubic operation, as the bladder is near the surface, being quite deep near the pubis. The tenaculum was avoided as the urine might be voided through the small opening. The buried silver wire suture was used owing to the fact that it took several weeks for the muscular tissue to unite, and no other suture would last that long. Without this suture according to the statistics of Gregg Smith 20 per cent. of cases of laparotomy had hernia. In the high operation it would make no difference if the peritoneum were cut. The wound in these cases was not left open as they were not cases of disease.

J. A. Goggans, of Alexander City, Ala., read a paper entitled,

EARLY DIAGNOSIS AND VAGINAL HYSTERECTOMY IN CANCER OF THE UTERUS, dwelling on the importance of prophylaxis, early diagnosis and early operative treatment by vaginal hysterectomy.

A. R. Robinson, of New York, read a paper on

THE TREATMENT OF MALIGNANT CUTANEOUS EPITHELIOMATA (CANCERS).

In cases where the diagnosis is not positive, iodide and mercury should be used. The elements in these cases extend much farther than is generally supposed. When they are cut out pathological cells are left, and we have so-called recurrence, which is a reappearance, when the wound has been treated antiseptically. It should be allowed to suppurate as the toxine of the pus is more destructive of the epithelioma cells than the erysipelas toxine. He opposed cutting and advocated caustics. The toxines had cured no cases but some may have been benefited. The caustic should destroy the tissue completely and quickly. Mild caustics as nitrate of silver should not be used.

Caustic potash liquifies the tissue as much as would be removed with the knife, beyond this there is inflammatory exudation which destroys the pathological elements. The cancer cells may extend deeper than could be reached with the knife; here the potash is preferable. There is less deformity than with the knife, more than with arsenious acid.

Chloride of zinc acts more slowly, suitable only in certain locations, as near the eye, also in the papillary form previous to the use of arsenious acid. Pain may be avoided by mixing it in twenty per çent. cocaine solution.

Arsenious acid has a more elective action on the cancer cells, and should as a rule be used weaker than Marsden's paste (2 to 1 part of gum Arabic). It may be used 3 to 2, or 1 to 1. This will not attack normal tissue in 20 hours. It should then be removed, and if not sufficient necrosis applied immediately for 16 to 18 hours. Cases should be watched for a year or two as there may be a reappearance. Arsenious acid may be applied to the lip if care is taken to prevent getting it into the mouth, but on the nose it is especially valuable on account of deformity.

These papers were discussed together.

R. R. Kime said that it was unfortunate that cancer of the uterus was not diagnosed early. The symptoms were sometimes not well defined, even when the disease was so far advanced that an operation is not advisable. Cases from 35 to 50 should be examined if any suspicion of cancer. If the general practitioner is in doubt he should refer the case to the gynecologist. If confined to the cervix this may be removed followed by caustics, the fatal results of hysterectomy being so much greater than in amputation of the cervix.

W. E. B. Davis thought that these cases were overlooked as they developed so insidiously. Every case of diseased cervix should be treated and cured. He advised removal of the uterus and ovaries for cancer.

P. L. Brouillette asked as to the danger of systemic effect from arsenious acid.

Dr. Goggans said that wherever there was cancer there had been irritation, hence the necessity of removing every source of irritation and every pathological condition as prophylactic.

Dr. Robinson had applied arsenious acid on a surface as large as the hand and had seen no systematic effect. Marsden advised not over an inch square. If seen early caustics are all that are necessary in cancers of the skin.

J. P. Stewart read a paper on

HEMORRHOIDS, in which he dwelt on the necessity of an examination under chloroform if necessary; where tumors are small he used an injection of red gum, if larger they are destroyed with clamp and cautery.

R. P. Johnson had treated cases successfully with injections of carbolic acid 2 parts to 1 of olive oil to which was added ¼ grain of morphine to each injection.

J. B. Cowan alluded to the new operation of excising all the hemorrhoidal tissue. These cases often fall into the hands of quacks who inject carbolic acid or ergot and temporarily relieve. He likes the old method of tying the tumors. In a bad case where there was no distinct tumor, he applied a method which was successful. This was to take up the tissue with a tenaculum and tying a part at a time. He thus put in fourteen ligatures. The result was perfect.

R. R. Kime dilates the sphincter and dissects the membrane from the tumor, which he ties if large. He called especial attention to the value of the knee chest position, thus ballooning the rectum.

G. A. Baxter insisted on not confining the treatment to any one method. Carbolic acid he considered dangerous. Could not examine without paralyzing the sphincter. He was always able to find a distinct tumor.

J. B. Murfree said it was better to dissect up the mucous membrane and ligate the vessels (after Allingham) than the whole tumor. Carbolic was valuable as a palliative and safe as a rule.

R. P. Johnson said that the piles are destroyed with carbolic acid if the solution is strong enough and the piles are filled.

Dr. Stewart said that carbolic acid was a complete failure.

R. R. Kime read a paper on

SYNTHETIC PERINEOTOMY IN LACERATIONS OF THE PERINEUM, using the term to designate a method of dividing and dissecting without loss of tissue. The redundancy is due to hyperplasia, a subinvolution, which will disappear when the cause (the laceration) is removed. The method was described in detail and cases related.

W. E. B. Davis said that there was more in the man being familiar with the operation he practiced than in any particular operation. It was important to differentiate between perineal tears and those of the posterior vaginal wall.

W. E. B. Davis, of Birmingham, read a paper on

BILE IN THE PERITONEAL CAVITY AND HOW TO DEAL WITH IT.

He presented an experimental and clinical study of the subject. The experiments confirmed the position taken in 1892 before the American Medical Association. The constant extravasation produced peritonitis unless there was satisfactory drainage. A considerable quantity would be walled off, just as any other irritating fluid. It was noted that in those cases where gauze was packed around the openings in the gall bladder or ducts that the animals recovered as a rule. The field of operation was walled off completely. Numbers were reopened in twenty-four to forty-eight hours and this condition found. In an operation on the human subject in which the gall bladder was removed and drainage with gauge and a glass tube, the field of the operation was completely walled off, and there was no evidence of general peritonitis. He took the position that in obstruction of the common duct for stone, that an incision should be made, the obstruction removed, and drainage established without an attempt being made to suture the opening, as these cases will not stand long operations as a rule.

R. J. Trippe, of Chattanooga, reported several cases of

APPENDICITIS, illustrating the necessity of early operation, and the fact that some will die, no matter when operated on, and also gave the technique.

These two papers were discussed together.

R. R. Kime said that authors differed as to time of operation. Many would get well anyhow, but it was difficult to tell which. Where a patient was improving, and got suddenly worse, operation is indicated, as it is probable that extravasation has taken place. When there is pus in the peritoneal cavity most operators advise against washing it out, but he would feel safer to flush the cavity and then drain.

C. Holtzclaw had had fifteen or sixteen cases of appendicitis, but had not been called to operate. They recovered with the ice pack over the inflamed area. Operation should be performed if any evidence of suppuration, elevation of temperature or collapse.

G. A. Baxter said that danger was not in operating but in delay. The question lies in the diagnosis between obstructive and catarrhal appendicitis. When we have a distinct enlargement and induration, we have obstruction.

R. M. Cunningham said that Dr. Davis was departing from his usual teaching, as he had said that normal bile would not produce peritonitis. In a large number of post mortems the appendix was not found diseased one time in a hundred. Operation is indicated where there is pus, swelling and induration.

J. P. Stewart always cures his cases of appendicitis with salines. He related a case of injury to the gall duct followed by distension of the gall bladder. He aspirated and got five pints of pus and bile, a few days later two pints, and again one pint; then the obstruction gave way and the same fluid passed per rectum.

J. B. Murfree said that no operation should be made for appendicitis until some indication, such as recurrence or gross local changes.

P. D. Sims could not recall a case of death from appendicitis which had not been operated on. It might be required, but swelling and hardness did not indicate an operation.

R. H. Hayes praised the high position taken by Dr. Davis in the surgery of the duct, and asked as to the preparatory and after-treatment.

In regard to appendicitis Telamon, of Paris, had published statistics giving 90 to 95 per cent. of cures without operation. An eminent English author gave out similar statistics, whether these are reliable or not he does not know. Many claim that when one has an attack of appendicitis, he has a constant source of danger in his belly. A few years back this was the main indication of operation, and in this view it seemed that the operation should be performed at any opportune time.

Dr. Davis thought that the case of Dr. Stewart was not a distension of the bladder, but that a cyst had formed around the bladder. It was saved by the aspiration. The after-treatment was the same as for other abdominal sections.

He thought catarrhal cases of appendicitis were common. When there is obstruction there is pain. Each case should be treated on its own merits. There are no hard and fast rules. Some needed operation. In general septic peritonitis the patient will die if operated on.

THIRD DAY.

J. R. Rathmell, of Chattanooga, read a paper entitled:

ACROMEGALY: REPORT OF A CASE.

He said that Paul Marie first described the disease in 1886, his theory being that enlargement of the pituitary gland was the cause of the disease. In the case reported with the symptoms common to this disorder, there were two uncommon symptoms. These were long continued abnormal rythm of the Cheyne-Stokes variety, and the inability to retain food or drink on his stomach for three months before. Both symptoms were accounted for by the enlarged pituitary body which weighed 475 grains instead of five or ten normally. The author believes the disease to be one of trophic origin, producing changes in the bony system, especially of the face, feet and hands, that the enlargement of the pituitary body and the other ductless glands was the result, not the cause of the disease.

W. C. Townes, of Chattanooga, read a paper on

ACROMEGALY, exhibiting specimens from Dr. Rathmell's case, the enlarged pituitary body, and a part of the ileum, with a diverticulum. He reported a case now under treatment, a marked feature of which was, that although fifty-two years old, there was no impairment of the sexual function. He believed the disease due to pressure on the brain produced by the enlarged pituitary body.

W. G. Bogart said these cases were rare. He had seen Dr. Rathmell's case. The enlarged tongue made articulation difficult. The breathing was labored and gave evidence of suffering. The points of interest were the length of time required for the development of the case fourteen years, and the question whether anything can be done if the diagnosis were made early.

E. A. Cobleigh asked if it were not possible that the condition was a persistent accentuation of a normal process, and if so, as to the cause. He reported a case presenting some of the symptoms.

J. Berrien Lindsley said that the paper was an evidence that the profession was advancing, and in this respect second to none. A disorder was being discussed the name of which had not yet got into the dictionaries.

J. R. Rathmell said that the first evidence of the disease was loss of strength and enlargement of extremities. He believes it a disease of the osseous system, especially of the feet, face and hands, but it affects the whole bony system, just as we have a pseudo-hypertrophic muscular paralysis. The line of treatment was to build up the system and electricity. He got some comfort from the latter.

J. B. Cowan, of Tullahoma, Tennessee, read a paper on

WATER VERSUS ATMOSPHERE THE CAUSE OF MALIGNANT MALARIAL FEVER,

And related observations where water from clear springs was evidently the cause of malarial fever. One case, where there were two springs connected underground, the upper was healthful, while those who used the lower had the disease. A marsh between the two formed a favorable nidus for the development of the miasm. In almost every instance where well and spring water had been abandoned for cistern water, malarial fever had disappeared. In a village supplied by a well all had the disease except one family that used other water. So many similar instances had come under his observation that he invariably changed the water in his malarial cases. If sterilized water were used malarial fever would be unknown.

E. T. Camp could not agree that malaria was taken in the water alone; some of the poison might be so absorbed, but it was said that in the tropics one would get a chill by sleeping in the open air. He believed it was taken mostly through the air.

C. Holtzclaw differed from Dr. Cowan, and related a case where there was no improvement by removing to a location where there had been no malaria for eight years.

W. G. Bogart said that water was one of the means of introducing the poison into the system, but not the only means. In a certain neighborhood where different waters were used all suffered alike, whether they were cleanly or not.

Y. L. Abernathy observed that when we have a wet summer we do not have much malarial fever. The paper seems conclusive that we can get the disorder from water, but why don't we have it every year, and why don't we have it all the time? The old theory was that it was due to heat, moisture and decomposition. Bowling's theory was that it was due to water confined so as to prevent evaporation. Now it is said to be due to a germ. Whichever is correct, it is true that nineteen-twentieths of the diseases of the West and South are due to malaria, so that we have to give quinine, iron and arsenic, etc.

Dr. Cowan said that the reason we did not have the disease every summer was because the heat did not develop the germ. All cases of malignant malarial fever were due to drinking water. In parts of the South where prevalent, and now unknown, it was due to the use of cisterns. In one case, a party camped several years on a lake, and had malaria; they escaped by taking their water with them, although sleeping in the open air, as before.

E. T. Camp, of Gadsden, Ala., read a paper on

### A COMPLICATED CASE OF OBSTETRICS WITH RUPTURE OF THE UTERUS.

It was a transverse presentation. The child was asphyxiated, but resuscitated. The placenta not being delivered in an hour, chloroform was administered, the hand introduced into the uterus, when the rupture was discovered. The placenta was adherent, but was torn away, morphine administered, but death took place in four hours. He thought rupture took place before the version, and was spontaneous. He thought if the placenta could have been gotten away, and contraction induced, the patient might have been saved.

Y. L. Abernathy thought this possible if a laparotomy had been performed.

W. G. Bogart said that the three interesting points were, 1st, the time of the rupture; 2d, the cause; 3d, the absence of symptoms. We would expect to have shock, with all the well marked conditions following. The strange thing is that there was no pain, loss of blood or shock. With the complications: malposition, adherent placenta and rupture of the uterus, with bad surroundings, death was inevitable.

J. P. Stewart thought the patient died from hemorrhage, not from shock. A post mortem would have been of interest.

D. S. Middleton thought the adherent placenta indicated a diseased structure, and this caused the rupture. The administration of chloroform opened the mouths of the vessels, and death was from hemorrhage.

C. Holtzclaw said that the only chance was to sew up the rent after doing a laparotomy.

J. R. Rathmell said that having a general adherent, fibroid placenta, the only thing to do was to let it alone and remove the entire uterus.

G. A. Baxter thought that an operation should have been done quickly, and the operation governed by circumstances after opening the abdomen.

D. M. Cunningham thought the doctor did the best to conserve his own interest. The case would have died any way. To be in line with modern surgery, an operation should have been performed.

Dr. Camp said there was nothing to indicate the condition until discovery was made. In a similar case, he would not operate, as the adherent placenta would have produced death later.

R. H. Hayes, Union Springs, Ala., read a paper on

### THE NUCLEINS AND THEIR RELATIVE POSITION IN THERAPEUTICS.

The nucleins are protoplastic or bioplastic cell substance, the bioplastic primal unit of the organism; the cell life, vital and resistant force; a proteid, granular cell life substance in which all vital energy and cell life resistant force and through which all animal nutrition took place. The nucleins are proteid bodies residing in the tissue cells and the yeast of certain plants (animal and yeast nucleins). The former taken from the blood and lymphoid glands of the body, residing principally in the polynuclear blood corpuscles of leucocytes, the proliferation of which they have the power of increasing (leucocytosis). They are natural defenders arresting and overwhelming all alien or disease germs as they enter the blood stream. Vaughan and McClintock have developed them.

They are gotten up in three forms, nuclein solution from the yeast of certain plants, vegetable; nuclein solution from the tissues of the body, thymus, thyroid, liver, spleen, etc., animal; and from the tissue direct, protonuclein. The principal difference between the antitoxines, is that the toxines antidote or antagonize a poison or ptomaine formed by the presence of alien or disease germs, and they belong to the class of albumen serum and attack the germs direct as soon as they reach the blood current. The nucleins are more direct, if less powerful, and have the advantage of attacking through the leucocytes any or all germs or poisons entering the system. Reports are encouraging from eminent men. The author reported an ulcer of sixteen years standing cured in four months. Another case of ulcer of the ankle (both nontubercular) very greatly relieved in same time. He favors, from limited experience, more general application of the nucleins.

G. W. Drake endorsed the use of these agents on theoretical grounds, and intends to use them and await results.

C. Holtzclaw cited the transfusion of blood as being a use of nucleins. Salt water does as well. The Valentine meat juice injected near an ulcer was found beneficial, but water does better. The whole business was an advertising scheme. These things should be under control of the government.

R. P. Johnson read a paper on

TREATMENT OF DIPHTHERIA, and gave his experience with established methods, endorsing especially the benzoate of soda. He read a letter from Prof. Klebs, who did not speak favorably of antitoxine, but claimed better results with antidiphtherine. He presented a plan to steam the patient with quicklime, by covering him with a sheet, to loosen the membrane.

G. A. Baxter said that the danger was from sepsis, and the germs were under the membrane, they could not be reached by local treatment. In a case treated here, the membrane liquified quickly under the serum treatment. Where death was from obstruction, which might extend to all parts of the lungs, no agent would liquify it quickly enough.

Geo. Thrash said that Prof. Klebs, in a paper read before the profession at Asheville, did not seem to be very enthusiastic about the serum treatment.

Y. L. Abernathy said that there were cases so mild as to need no doctor, and some so bad as to need no doctor. The membrane is not dangerous until it gets into the trachea, and then the case generally dies. Tracheotomy is all that promises anything, and it is generally a failure.

J. Berrien Lindsley presented a paper on

PREVENTION OF SMALL-POX.

He did not believe one-fourth of the people of Tennessee were vaccinated. Doctors were not willing to acknowledge their inability to diagnose small-pox, hence mistakes occur. In Little Rock, the disease was under way six weeks before its nature was discovered. A rule of sanitary boards is, in case of doubt, to give the public the benefit of the doubt, and isolate the case. All suspicious cases should be sent to some institution. He related several cases where undiagnosed small-pox spread, resulting in death.

H. Berlin said the micro-organism of the disease had not been discovered, and we were left to empiricism. Jenner had done more for the race than Napoleon in all his wars. In the Franco-Prussian war the French lost more from small-pox than were killed in battle; small-pox was unknown in the German army, as the soldiers had been vaccinated.

R. P. Johnson did not think the disease very contagious until after pustules were formed. Danger increases with desquamations. Physicians should have their patrons vaccinated.

Dr. Lindsley said that the reason small-pox was dangerous was because of the neglect of vaccination. The fear of it did not formerly exist. The profession had not done its duty in the matter, failing to insist on its importance.

The following officers were elected for the ensuing year:

President, J. B. Murfree, Murfreesboro, Tenn.

Vice-Presidents, R. J. Trippe, Chattanooga; R. R. Kime, Atlanta; R. H. Hayes, Union City, Ala.

Secretary, Frank Trester Smith, Chattanooga.

Treasurer, G. R. West.

The next meeting will be held in Nashville, October 13, 14 and 15, 1896.

The following were read by title:

"When Consumptives Should Go to Colorado, and Why," J. C. Minor; "Diagnosis of Incipient Phthisis," L. P. Barbour; "Some Simple Procedures in Tedious Labor," R. M. Harbin; "Sympathetic Ophthalmia," Frank Trester Smith.

